# Prevalence of kidney disease and associated factors among adults with chronic hepatitis B virus in Southwestern Uganda

**DOI:** 10.1097/MS9.0000000000004402

**Published:** 2025-11-24

**Authors:** Samuel Jjunju, Edwin Nuwagira, Mike Ssemusu, Grace Kansiime, Conrad Muzoora

**Affiliations:** a Department of Internal Medicine, Mbarara University of Science and Technology, Mbarara, Uganda; b Department of Internal Medicine, Soroti University, Soroti, Uganda; c Department of Internal Medicine, Mbarara Regional Referral Hospital, Mbarara, Uganda

**Keywords:** chronic hepatitis B virus, kidney disease, neutrophil gelatinase–associated lipocalin

## Abstract

**Background:**

Hepatitis B virus (HBV) is a global health problem endemic in Sub-Saharan Africa, with 65% of global cases occurring in the WHO Western Pacific and African regions. HBV is a known primary cause of kidney disease that progresses throughout the illness. Early detection of kidney disease and antiviral initiation can slow its progression. Our study aimed to identify the prevalence of kidney disease and associated factors in adults with chronic HBV.

**Methods:**

We conducted a cross-sectional study from November 2023 to December 2024 in Mbarara, Uganda, recruiting adults with chronic hepatitis B. Data collected: socio-demographics, comorbidities, coinfections, and hepatitis B-related data. Spot urine and blood were collected for dipstick, urine neutrophil gelatinase–associated lipocalin (NGAL), and creatinine with estimated glomerular filtration rate (eGFR). We defined kidney disease as the presence of (1) urine NGAL >132 ng/mL, (2) eGFR <60 mL/min/1.73 m^2^, or (3) eGFR between 60 and <90 mL/min/1.73 m^2^ with proteinuria or hematuria. We utilized logistic regression to determine factors associated with kidney disease.

**Results:**

We enrolled 157 participants with a mean age of 35.8 (standard deviation, ±12.5) years, and 50% (79/156) were males. Overall kidney disease prevalence was 27% (43/157; 95% confidence interval (CI): 20.6–35.1%). Prevalence based on urine NGAL was 17.2% (27/157; 95% CI: 11.6–24.0), eGFR <60 mL/min/1.73 m^2^ was 5.7% (9/157; 95% CI: 2.7–10.6), and eGFR between 60 and <90 mL/min/1.73 m^2^ with either proteinuria or hematuria was 9.6% (15/157; 95% CI: 5.4–15.3). Being female was associated with higher odds of kidney disease (adjusted odds ratio, 2.49; 95% CI: 1.16–5.36; *P* = 0.019).

**Conclusion:**

Nearly one-third of adults with chronic Hepatitis B in Uganda had kidney disease, with NGAL increasing the detection by 12% over creatinine and urine dipstick alone. We recommend frequent monitoring of kidney function in adults with chronic HBV.

## Introduction

Hepatitis B virus (HBV) infection remains a significant global health threat with an estimated 254 million persons worldwide predicted to have chronic HBV infection in 2022, with 65% of cases occurring in the Western Pacific and African regions^[1]^. The estimated prevalence of HBV in Uganda was 4.3% in Uganda in 2019^[2]^. A hospital-based cross-sectional study among patients in southwestern Uganda found a prevalence of HBV at 5.5%^[3]^.

**HIGHLIGHTS**

- Hepatitis B virus (HBV) is a global health problem and a primary cause of kidney disease.
- Our study findings show that nearly one-third (27%) of adults (especially females) with chronic HBV in Uganda have kidney disease.
- Urine neutrophil gelatinase–associated lipocalin increases the detection by 12% over creatinine and urine dipstick alone.
- According to our findings, we recommend frequent monitoring of kidney function in adults with chronic HBV.

HBV infection is a known primary cause of kidney disease^[4]^. Numerous mechanisms have been proposed for the development of kidney disease from HBV and these include the deposition of immune complexes in the glomerulus and small and medium blood vessels to form glomerulonephritis and vasculitis, respectively^[5]^.

Membranous and membranoproliferative are the most common types of glomerulonephritis in adults with HBV, while mixed cryoglobulinemia and polyarteritis nodosa are the most common vasculitides that these patients develop^[5]^. Other mechanisms include induction of kidney tubular cell injury and apoptosis by triggering a pathway of Fas up-regulation^[6]^ and the nephrotoxic effect of antiviral medications such as tenofovir disoproxil fumarate (TDF)^[7]^, in addition to comorbidities like hypertension, diabetes mellitus, human immunodeficiency virus (HIV), and hepatitis C co-infection^[8]^.

With the global increase in the burden of kidney disease, it is estimated that 850 million people suffer from kidney disease currently making it the 12th highest cause of death worldwide, with most cases occurring in Sub-Saharan Africa^[9]^. The general prevalence of kidney disease is estimated to be at 13.9% in Africa and as high as 21.4% in Uganda, making it one of the primary contributors to morbidity and mortality^[10]^.

Other than the standard risk factors for kidney disease, such as hypertension, diabetes mellitus, HIV, smoking, and alcohol use, HBV is increasing as a major cause of kidney disease^[11]^. A study conducted on the general population in Uganda demonstrated that two-thirds of individuals with kidney disease do not have the traditional risk factors, which include HIV infection, diabetes mellitus, and hypertension^[10,12]^. In addition, a significant number of patients with kidney disease in Uganda delay seeking care, with 51% presenting with end-stage kidney disease (ESKD) at their first visit, which makes diagnosis of the underlying cause impossible^[13]^.

Although the prevalence of chronic HBV infection is high in Africa, little is known about the prevalence of HBV-associated kidney disease. In a study using combined urine albumin to creatinine ratio or estimated glomerular filtration rate (eGFR) in patients receiving anti-HBV antiviral treatment in rural China, the prevalence of kidney disease was 7.9%^[14]^. This Chinese study found cirrhosis, diabetes mellitus, and hypertension as significant factors associated with the development of kidney disease^[14]^. Using the eGFR and other indicators of kidney damage such as proteinuria, hematuria, and glycosuria, a French study found that 65% of those with chronic HBV had renal abnormalities. Whereas using the eGFR criteria alone, a study in Cameroon found that patients with chronic HBV had a kidney disease prevalence of 20%, with age ≥50 years, chronic use of herbal drugs, and HBV known duration for ≥5 years being all associated with kidney disease^[15]^.

The majority of studies have used serum creatinine–based eGFR to assess kidney function in patients with chronic HBV despite its significant drawbacks^[16]^. Serum creatinine has a poor sensitivity (77%), but a great specificity (91%), especially in the early phases of kidney disease^[17]^. Scientists have looked into more recent novel kidney function biomarkers, including urine neutrophil gelatinase–associated lipocalin (NGAL), with a sensitivity range of 90–93% and a specificity of 71–99%^[18,19]^ in detecting early changes in kidney function^[20,21]^.

In patients with chronic HBV, using kidney function biomarkers like urine NGAL would help in the detection of early kidney disease, so that the timely introduction of HBV treatment can be effected to delay the progression to ESKD^[22,23]^. In adults, HBV-associated kidney disease is linked with the progression of kidney disease to ESKD, necessitating kidney replacement therapy if no treatment is given^[4]^.

Our study aimed to determine the prevalence of kidney disease and associated factors among adults with chronic HBV using a combination of kidney function biomarkers including a novel biomarker, urine NGAL, in a cohort of adults with chronic HBV in southwestern Uganda. This cross-sectional study has been reported in line with the STROCSS guidelines^[24]^.

## Methods

### Study design

This was a cross-sectional study carried out from November 2023 to December 2024.

### Participants and setting

We prospectively enrolled participants with chronic HBV attending hepatitis clinic in a tertiary hospital in Mbarara, which is located in the southwestern part of Uganda.

In this clinic, chronic HBV infection is defined as the persistence of hepatitis B surface antigen for 6 months or more after acute infection. Criteria for starting antiviral treatment for hepatitis B include the following: liver cirrhosis, alanine aminotransferase (AST) to platelet ratio index score >2, HIV co-infection, persistent elevated alanine aminotransferase (ALT) > 40 U/L (measured on three occasions at least every 3 months over a period of 6–12 months), or hepatitis B viral load >20,000 IU/mL. Patients who meet the criteria for antiviral treatment are started on TDF. Other antiviral agents like tenofovir alafenamide and entecavir are not readily available in southwestern Uganda.

At the time of this study, the total number of patients under care for chronic HBV was 350, and all were considered for inclusion in our study during the entire study period. We used the finite population formula to calculate our sample size. Using a study from Cameroon where kidney disease prevalence was 18.4% of chronic HBV patients and a finite population of 350 patients, it gave us a sample size of 139 participants. However, we ended up consecutively enrolling 157 consenting participants after excluding pregnant mothers, breastfeeding mothers during the first 6 months of the postpartum period, current menstruation, and confirmed urinary tract infection due to the potential effect on our urinary measurements of hematuria and proteinuria.

The eGFR was calculated using the Chronic Kidney Disease Epidemiology Collaboration (CKD EPI) 2021 equation, which incorporates serum creatinine, age, and sex^[25]^. We defined kidney disease as the presence of any of the following: (1) raised urine NGAL >131.7 ng/mL, (2) eGFR <60 mL/min/1.73 m^2^, or (3) eGFR between 60 mL/min/1.73 m^2^ and <90 mL/min/1.73 m^2^ with the presence of proteinuria or hematuria. The cutoff point threshold for urine NGAL of >131.7 ng/mL was based on the manufacturer’s definition of kidney disease; however, it has never been validated in the African or chronic HBV population.

### Study procedures

Eligible participants were selected by consecutive sampling and a written informed consent form was obtained. We used a pre-tested questionnaire to collect participant demographics and clinical data, including HIV and hepatitis C status, history of smoking and alcohol use, Hepatitis B envelope antigen status, history of antiviral medication, herbal medicines, history of hypertension, and diabetes mellitus. Body mass index was calculated from current height and weight measurements performed using a standardized weighing scale and stadiometer. Biological specimens (blood and urine) were taken off by trained staff and transferred to the clinical laboratory to determine serum creatinine and liver enzymes (ALT and AST) using a fully automated chemistry analyzer (Humastar 200, Human Diagnostics, Wiesbaden, Germany). Complete blood counts were obtained using a fully automated hematology analyzer (Sysmex XN 550, Sysmex corporation, Kobe, Japan), and urine NGAL was measured using a finecare FIA meter plus machine (Guangzhou Wondfo Biotech Company limited, Guangzhou, China) and a urine dipstick test (uristix, 10 parameter, Cypress Diagnostics, Hulshout, Belgium). The laboratory performs daily internal quality control checks on all the equipment machines before working on any sample.

### Statistical analysis

The data were transferred into an Excel spreadsheet and then exported to STATA version 17 (StataCorp, Texas, USA) for analysis. Categorical variables were reported as frequencies and percentages, whereas continuous variables were reported as mean and standard deviation (SD) or in the case of skewed data, median and interquartile ranges (IQR). The prevalence was reported as simple proportions with a 95% confidence interval (CI). We used a logistic regression model (crude and adjusted) to identify the factors associated with kidney disease, and a *P*-value of <0.05 was considered significant. In bivariate analysis, factors with a *P*-value of <0.3 and those known to be associated with kidney disease were included in the multivariate analysis.

### Ethical considerations

All study participants gave written informed consent to participate in the study. The study was carried out in adherence to the Declaration of Helsinki. All datasets used in this analysis may be shared through direct contact with the corresponding author on reasonable request.

## Results

We screened 175 adults with a diagnosis of chronic HBV from November 2023 to December 2024. We excluded 18 patients because of pregnancy (5), active menstruation (2), and a decline to consent (11). The mean age was 35.8 (SD: ±12.5) years, and 50% were men (79/157). The majority of participants, 26.8% (42/157), were farmers and residing in Mbarara district, 31.9% (50/157). We found 5.1% (8/157) had a history of smoking, and only 19.1% (30/157) had a history of alcohol use. Only 1.3% (2/157) had HIV coinfection, 3.2% (5/157) had hepatitis C, 10.8% (17/157) had a history of hypertension, and none had diabetes mellitus (Table 1).

**Table 1:** Baseline characteristics of participants with chronic hepatitis B virus infection

Variable	Total (*N* = 157)
Age, years (mean ± SD)	35.8 ± 12.5
Body mass index, kg/m^2^ (mean ± SD)	23.4 ± 4.5
Male sex, *n* (%)	79 (50.3%)
Married, *n* (%)	106 (67.5%)
Mbarara district, *n* (%)	50 (31.9%)
Occupation of farming, *n* (%)	42 (26.8%)
History of smoking, *n* (%)	8 (5.1%)
History of alcohol intake, *n* (%)	30 (19.1%)
Hypertension, *n* (%)	17 (10.8%)
HIV co-infection, *n* (%)	2 (1.3%)
Hepatitis C co-infection, *n* (%)	5 (3.2%)
Duration since diagnosis of hepatitis B, years (median, IQR)	1.6 (0.8–4)
Tenofovir disoproxil fumarate use, *n* (%)	63 (40.1%)
History of herbal medicine use, *n* (%)	48 (30.6%)
Duration of use (months) (median, IQR)	2 (1–12)
ALT, U/L (median, IQR)	25 (20–36)
AST, U/L (median, IQR)	27 (22–35)
Hepatitis B viral load (IU/mL) (median, IQR)	20 (0–4060)
HBeAg positive, *n* (%)	37 (23.6%)
Platelet ×10^3^/µL (mean ± SD)	254 ± 95.4
Hemoglobin, g/dL (mean ± SD)	13.7 ± 1.8
White blood cells ×10^3^/µL (mean ± SD)	6.6 ± 1.8
AST to platelet ratio index score (median, IQR)	0.3 (0.3–0.3)

ALT, alanine transaminase, AST, aspartate transaminase, HBeAg, hepatitis B virus envelope antigen, HIV, human immunodeficiency virus, IQR, interquartile range, SD, standard deviation.

Our study found an overall prevalence of 27.4% (43/157) with a 95% CI of 20.6–35.1 when using any of the following parameters: urine NGAL, eGFR <60 mL/min/1.73 m^2^ or eGFR between 60 mL/min/1.73 m^2^ and <90 mL/min/1.73 m^2^ with either hematuria or proteinuria. Using the individual biomarkers for kidney function, urine NGAL gave the highest prevalence of 17.2% (27/157, 95% C.I: 11.6–24.0) compared to eGFR <60 mL/min/1.73 m^2^ based on serum creatinine at 5.7% (9/157, 95% C.I: 2.7–10.6) or eGFR between 60 and <90 mL/min/1.73 m^2^ with either proteinuria or hematuria and 9.6% (15/157, 95% C.I: 5.4–15.3; Table 2).

**Table 2:** Prevalence of kidney disease among patients with chronic hepatitis B virus infection

Prevalence of kidney disease	Frequency (*N* = 157)	Percentage	95% CI
Overall prevalence	43	27.4%	20.6–35.1
Urine NGAL elevation	27	17.2%	11.6–24
eGFR <60 mL/min/1.73 m^2^	9	5.7%	2.7–10.6
eGFR between 60 and <90 mL/min/1.73m^2^ and proteinuria or hematuria	15	9.6%	5.4–15.3

eGFR, estimated glomerular filtration rate, NGAL, neutrophil gelatinase–associated lipocalin.

In multivariate analysis (Table 3), only female sex was found to be significant with adjusted odds ratio (AOR) of 2.49 (95%CI: 1.16–5.36; *P* = 0.019). Patients who were on TDF medication were 1.58 times more likely to develop kidney disease, but with a nonsignificant *P*-value (95% CI: 0.73–3.42; *P* = 0.247). Those who had HIV coinfection were 3.6 times more likely to develop kidney disease, but with a nonsignificant *P* value and very wide CI (95% CI: 0.2–63.79; *P* = 0.381). Patients who had hepatitis C coinfection were 1.56 times more likely to develop kidney disease, but with a nonsignificant *P*-value and wide CI (95% CI: 0.0.23–10.64; *P* = 0.652).

**Table 3:** **Univariate and multivariate logistic regression analysis for factors associated with kidney disease among patients with chronic hepatitis** **B virus infection**

	Univariate analysis		Multivariate analysis	
Variable	Crude odds ratios (95% CI)	*P*-value	Adjusted odds ratio (95% CI)	*P*-value
Age >40 years	1.38 (0.62–3.07)	0.432		
History of alcohol intake	0.77 (0.30–1.95)	0.580		
History of smoking	0.88 (0.17–4.53)	0.877		
Underweight (BMI <18.5 kg/m^2^)	1.26 (0.45–3.56)	0.663		
Overweight (BMI >25 kg/m^2^)	0.83 (0.39–1.78)	0.637		
Duration since diagnosis of hepatitis B >3 years	1.19 (0.58–2.43)	0.635		
Hepatitis B viral load ≥20 000 IU/mL	1.09(0.45–2.65)	0.852		
Fibrosis APRI score (≥0.5)	1.11 (0.49–2.49)	0.805		
HBeAg positive	0.81 (0.35–1.90)	0.633		
Tenofovir disoproxil fumarate use	1.64 (0.81–3.32)	0.173	1.58(0.73–3.42)	0.247
Hepatitis C coinfection	1.80 (0.29–11.19)	0.526	1.56(0.23–10.64)	0.652
HIV coinfection	2.69 (0.16–43.99)	0.488	3.61 (0.20–63.79)	0.381
Hypertension	2.67 (0.96–7.44)	0.061	2.10 (0.70–6.26)	0.183
Female sex	2.39 (1.15–4.95)	**0.019**	2.49 (1.16–5.36)	**0.019**
Herbal medicine use	1.52 (0.72–3.19)	0.269	1.39 (0.64–3.02)	0.407

APRI, aspartate transaminase to platelet ratio index, BMI, body mass index, CI, confidence interval, HIV, human immunodeficiency virus, HBeAg, hepatitis B virus envelope antigen.

Bold values are the statistically significant values at both univariate and multivariate analysis.

## Discussion

Our study found an overall kidney disease prevalence of 27.4% (95% CI: 20.6–35.1%) when using any of three study parameters (urine NGAL, eGFR <60 mL/min/1.73 m^2^, and eGFR between 60 and <90 mL/min/1.73 m^2^ with either hematuria or proteinuria). When using the individual kidney function biomarkers, urine NGAL gave a higher prevalence of 17.2% (95% CI: 11.6–24.0) compared to eGFR <60 mL/min/1.73 m^2^ of 5.7% (95% CI: 2.7–10.6) while combined urine dipstick abnormalities with eGFR between 60 and <90 mL/min/1.73 m^2^ gave a prevalence of 9.6% (95% CI: 5.4–15.3). Adding urine NGAL testing increased detection by 12%.

Both chronic HBV and kidney disease pose a great health problem, especially in low- and middle-income countries globally^[1,9]^. Our study found a lower overall prevalence of kidney disease than the HARPE study done in France among antiretroviral naïve patients with HBV (27.4% vs 64.6%)^[8]^. We think the HARPE study found a higher prevalence because of the definition of kidney disease where they used GFR <60 mL/min/1.73 m^2^, GFR between 60 and 89 mL/min/1.73 m^2^ with kidney damage markers on urinalysis, and lastly normal GFR (≥90 mL/min/1.73 m^2^) with kidney damage markers on urinalysis^[8]^.

However, our overall prevalence was relatively higher compared to another study that was done in Cameroon among patients with chronic HBV, which found an overall kidney disease prevalence of 19.9% and 18.4% when using CKD-EPI and MDRD formula, respectively^[15]^. Another hospital-based cross-sectional study done in China also found a lower prevalence of 7.9% when using the CKD-EPI formula GFR <60 mL/min/1.73 m^2^^[15]^. We believe the major cause of this discrepancy is that our study used a novel kidney function biomarker with greater sensitivity and specificity for kidney disease, especially in the early stages^[18,19]^.

When basing on urine NGAL to assess for kidney disease, we found a higher prevalence (17.2%) compared to 5.7% when basing on eGFR <60 mL/min/1.73 m^2^ alone, which was almost similar to the study conducted in China^[14]^. This shows that urine NGAL can detect a greater percentage of kidney disease among patients with HBV, especially in earlier stages, compared to serum creatinine.

Initially, urine NGAL was mostly used for early detection of acute kidney injury and acute kidney disease as it would rise early before changes in serum creatinine or GFR^[18,20,26]^. However, recent studies have shown that urine NGAL rises in the early stages of CKD as well and can be used to predict the progression of CKD^[19,27]^. The higher sensitivity of urine NGAL may explain the high prevalence of kidney disease in our study compared to studies that used eGFR alone.

Our study found that being female was the only associated factor for the development of kidney disease among patients with chronic HBV infection in our setting with an AOR of 2.49 (95% CI: 1.16–5.36, *P* = 0.019). Some of the suggested hypotheses include females being more prone to urinary tract infections, which cause kidney damage, and also women may be more prone to suffer kidney disease as a result of complications during pregnancy, such as hypertension or eclampsia, that put women at higher risk of kidney injury^[28,29]^. Our findings are similar to other studies done in the general population in Uganda where female sex was one of the associated factors for kidney disease^[10]^. Compared to the study by Patrice *et al* in Cameroon, which found that older age, longer duration of HBV infection, and chronic use of herbal medicine were strongly associated with kidney disease^[15]^, our study had a predominantly young population with relatively short duration (<4 years) since the diagnosis of HBV. While some of our participants reported use of herbal medication, this was not statistically significant in our study compared to the Cameroon study. This may be because the two populations are taking different herbs with differences in their nephrotoxicity.

Compared to most studies^[8,14,30]^, we did not find an association between the known traditional risk factors for kidney disease, including hypertension, diabetes mellitus, liver cirrhosis, HIV, and hepatitis C coinfection in our study. This may be partly because of the small sample size, and also few participants in our study had these comorbidities/coinfections.

This is the first study to evaluate kidney disease prevalence among patients with chronic HBV in Uganda, and it is the first study to assess the use of novel biomarkers (urine NGAL) in patients with HBV in Africa. Due to the overall small number of participants, our study was generally underpowered to conclusively evaluate the factors associated with kidney disease.

In summary, our study found a high overall prevalence of kidney disease among patients with chronic HBV at 27.4%. Using the novel urine NGAL biomarker increased the prevalence of kidney disease compared to eGFR alone or eGFR plus proteinuria/hematuria. Our study also found that females with HBV have a higher likelihood of having kidney disease compared to males.

We recommend regular kidney function monitoring for patients with chronic HBV, more specifically among women. We recommend an implementation science study to evaluate the use of urine NGAL in screening for kidney disease among adults with HBV attending hepatitis clinics in Uganda to assess its acceptability, cost-effectiveness, and accuracy against the standard in our setting.

## Data Availability

All datasets used in this analysis may be shared through direct contact with the corresponding author on reasonable request. By recommendation of the Uganda National Council of Science and Technology, it is required to explain the aim of the requested information. The information will be shared while respecting the confidentiality of the patients included in the study.

## Research registration unique identifying number (UIN)

The study was registered under the Uganda National Council of Science and Technology, registration ID HS4186ES.

## Assistance with the study

We acknowledge study team members: Bobson Natamba, Jackline Nakanwagi, Susan Nimusiima, Benjamin Mwine Bigirwa, staff, and the patients at the hepatitis clinic of Mbarara Regional Referral Hospital. We also acknowledge Prof. David Meya and Prof. David Boulware for their support in the manuscript review.

## Presentation

We presented an abstract at the 2025 International Society of Nephrology, World Congress of Nephrology in New Delhi, India.
